# Working for Long Hours Is Associated With Dietary Fiber Insufficiency

**DOI:** 10.3389/fnut.2022.786569

**Published:** 2022-02-18

**Authors:** Jeehee Min, Dong-Wook Lee, Mo-Yeol Kang, Jun-Pyo Myong, Hyoung-Ryoul Kim, Jongin Lee

**Affiliations:** ^1^Department of Occupational and Environmental Medicine, Seoul St. Mary's Hospital, College of Medicine, The Catholic University of Korea, Seoul, South Korea; ^2^Department of Preventive Medicine, College of Medicine, Seoul National University, Seoul, South Korea

**Keywords:** dietary fiber (DF), feeding behavior (MeSH), long work hours, occupational health, nutrition

## Abstract

It has been suggested that long working hours are associated with various diseases through dietary patterns. However, few studies have reported the association between working hours and dietary habits. Thus, the objective of this cross-sectional study was to explore the difference in dietary fiber intake by working hours. Data of a total of 10,760 workers in South Korea who participated in the Korea National Health and Nutrition Survey (KNHANES), a nation-wide survey, were analyzed to determine different distributions of dietary fiber insufficiency using multiple logistic regression models. Fiber insufficiency proportion was different from working hour groups. 70.1% of the total population eat fiber insufficiently. Working <40 h group showed 66.8% of fiber insufficiency. But working more than 52 h group marked 73.2% of fiber insufficiency. Logistic regression analysis of 10,760 nation-wide study participants revealed that working for 41–52 h a week (OR: 1.32, 95% CI: 1.18–1.47) and working for over 52 h a week (OR: 1.42, 95% CI: 1.25–1.62) were significantly associated with insufficient fiber intake compared to workers with standard working hours (30–40 h a week). These associations were still robust in an adjusted model, with working for 41–52 h a week (OR: 1.13, 95% CI: 1.01–1.27) and working for over 52 h (OR: 1.26, 95% CI: 1.09–1.45) showing high associations with dietary fiber insufficiency. Those with long working hours tend to have insufficient intake of dietary fiber. To promote desirable dietary habits, intervention programs on working conditions should be considered.

## Introduction

Diet is an important factor that affects human health. Many evidence-based studies have shown that taking fruits, non-starchy vegetables, and minimally processed whole grains can prevent cardiovascular diseases (1). Foods with priority for health generally contain high contents of dietary fibers, which are plant-derived materials that are resistant to digestion by human alimentary enzymes (2). They are associated with decreased risk of a number of diseases by improving lipid profiles and inflammatory markers (3).

Long working hours are closely associated with various adverse health effects. A a series of meta-analyses and systematic reviews have consistently shown that long working hours can increase the risk of cardiovascular diseases (4–6). The mechanism by which long working hours increase the risk of cardiovascular diseases can be explained by the association between long working hours and the incidence of hypertension or diabetes mellitus (7). Long working hours are also associated with poor disease control in patients diagnosed with diabetes mellitus (8).

However, two studies with a longitudinal design conducted in Japan did not show any significant relation of long working hours with hypertension or diabetes mellitus (9, 10). In a large meta-analysis, the association between diabetes mellitus and long working hours was limited to groups with a low socioeconomic status (11). These findings suggest that biological mechanisms could not solely explain the increased risk of cardiovascular disease by working for long hours. Psychosocial and/or behavioral mechanisms should also be considered (12). Physical activity is a good example because there are irrefutable evidences that physical activity can prevent adverse health conditions including cardiovascular diseases (13). As long working hours are associated with subjective symptoms such as fatigue and headache, people with long working hours tend to spend less time in physical activity (14, 15). Furthermore, people with long working hours are more likely to increase their alcohol use (16). A recent systematic review has shown that those who have long working hours are at increased risk of obesity, which might be a result of unhealthy behaviors and/or psychosocial factors (17, 18).

A previous study has also shown that younger workers with long hours and shift work schedule do not have sufficient intake of micronutrients with under-intake of total energy and nutrition (19). As described above, many previous studies have focused on behavioral changes caused by long working hours. However, dietary behaviors were not dealt with. Considering that long working hours are known to cause behavioral changes, differences in dietary behaviors between people who work for long hours and those who do not work for long hours are expected. Thus, the objective of this study was to explore differences in dietary fiber intake by long working hours.

## Subjects and Methods

### Study Participants

This study used the Korean National Health and Nutrition Examination survey (KNHANES), which was conducted by Korea Centers for Disease Control and Prevention. KNHANES is an annual cross-sectional survey to determine participants' health status and nutrient status representing the entire Korean population. This survey consisted of health examinations, dietary interviews, and laboratory tests. Study participants were selected by stratification of geographical location, housing type, sex, age, and education level to represent the national population. We used KNHANES data from 2013 to 2019 (7 years) when the survey contained information on dietary fiber intake. As a result, 49,089 participants were included initially. We limited study participants to a working population. People with any missing values for important variables (e.g., dietary information, working hours) were excluded. Moreover, we excluded participants with working hours for <30 h per week (not full-time workers) to assure study eligibility (20). As a result, 10,760 participants were eligible for this study.

### Demographic Characteristics

In KNHANES, demographic characteristics were collected using a questionnaire. We collected information on sex, age, household income, education level, body mass index (BMI), alcohol drinking, and smoking status. Occupational groups were divided into two groups: manual workers (agriculture, forestry and fisheries workers, craft, device and machine operators, assembly workers, and simple laborer) and non-manual workers (managers and professionals, office workers, and service and sales workers) (21). Employment status was categorized into three groups: full-time employment, temporary job, and day labor. Shiftwork status was categorized into two groups: shiftwork and non-shiftwork. The shiftwork group consisted of all types of working types except day work, such as three shifts, permanent night work, evening shift work, 24-h shifts, and so on.

### Working Hours

Working hour was asked with the following question: “What is the average weekly working hours at work, including overtime? (Eating time is excluded)”. According to Korean Labor standards Act Article 50, statutory working hours per week should be limited to within 40 h per week. Working for up to 52 h per week is allowed by overtime or holiday works. Working for more than 52 h per week is illegal basically. However, it is allowed for some special occupational groups, for example, health care workers. People who worked for <30 h were excluded from this study. Therefore, working hours per week were categorized into three groups: (1) 40 h or shorter (30–40 h), (2) from 41 to 52 h (41–52 h), and (3) longer than 52 h (>52 h).

### Dietary Fiber Intake

Nutrition intake was surveyed using the 1-day (24-h) recall food survey method. A nutrition survey was executed with face-to-face interviews by trained surveyors. Study participants were asked to recall all kinds and portions of food and beverage. Even the same food was classified according to whether it was cooked as a school meal, at home or at a restaurant. According to the predetermined recipe database (DB) conducted by KNHANES, consumed food was converted to ingredients and mass. And according to predetermined food nutritional ingredient DB, each ingredient was calculated as a nutrient profile. Nutrient information was calculated according to the predetermined recipe DB (22). Total energy and fiber intake was used in this study. Fiber intake per daily energy intake, which was daily consumed fiber (g) divided by daily energy intake (kcal), was then calculated. Fiber insufficiency was defined as daily fiber intake <14 g/1,000 kcal (23).

### Statistical Analysis

Demographic characteristics, which were categorical variables, were compared among the three working hour groups using Chi-squared tests. Because KNHANES was extracted and stratified with a multi-stage clustered design, data were analyzed considering survey weights. KNHANES is provided with a guideline for data analysis in which deals with proper selection of weight variable by different survey sectors. The guideline is accessible in English version of the KNHANES webpage: https://knhanes.kdca.go.kr/knhanes/eng/.

For calculating the proportion and means of demographic variables, a survey weight named “wt_itvex” was used because the variables are from basic questionnaires. Daily fiber intake was compared among the three working hour groups using analysis of variance (ANOVA). Fiber amount (grams) per daily intake energy was also compared among the three working hour groups using ANOVA. Fiber insufficiency rate was calculated and compared among the three working hour group with ANOVA. For comparing daily fiber intake by working hour groups, we used a weight variable “wt_ntr” because daily fiber intake was calculated from nutrition survey data.

Odds ratios (ORs) and 95% confidence intervals (95% CIs) of fiber insufficiency rate in long working groups were calculated with logistic regression analysis models. At first, simple logistic regression analyses were done by sex, age, BMI group, occupation category, employment status, drinking habit, household income, education level, and shiftwork. Afterward, multiple logistic regression analysis was done after adjusting for covariates such as sex, age group, occupation category, drinking and smoking habit, and education level, which had significant associations with dietary fiber insufficiency in simple logistic regression models. A weight variable “wt_tot” was used for comparing variables from both basic questionnaires and nutritional survey.

Age was stratified into five groups (the 20s, 30s, 40s, 50s, and 60s or older) because fiber intake was significantly different by age groups. Simple logistic regression analysis and multiple logistic regression analysis were done in the same manner as the logistic regression models above.

All the statistical analyses were performed with R program version 4.1.1 We used an R package “survey” to apply survey weights in each step of the analysis. After calling the package with a command “library”, we declared the survey model with a command “svydesign” in the survey package with appropriate identification numbers and survey weight variables in each step. Chi-square analyses were using a command “svychisq” with the declared survey design. The results from ANOVA were called from a command “anova” after applying “svyglm” in association with daily fiber intake and dependent variables. In logistic regression analyses, svyglm with an option of “family = binomial” was used.

## Results

Among a total of 10,760 study participants, 4,973 (46.2%), 3,694 (34.3%), and 2,093 (19.5%) worked for <40 h a week, 41–52 h a week, and more than 52 h a week, respectively. Demographic characteristics showed statistically significant differences by weekly working hours. More women (54.8%) worked for <40 h per week and more men (70.2%) worked for more than 50 h per week. The proportion of overweight was the largest in the group working for more than 52 h a week (38.9%). The proportion of those with drinking habits was the largest in the group working for more than 52 h per week (23.8%). Smoking habit was also more prevalent in the group working for more than 52 h per week (34.5%). Household income tended to be lower in the group working for more than 52 h per week (Table 1).

**Table 1:** Demographic characteristics of study participants.

	**Total**	**Weekly working hours**			***P*** **-value**
		**30–40 h**	**41–52 h**	**>52 h**	
	***N*** **(%)**	***N*** **(%)**	***N*** **(%)**	***N*** **(%)**	
**Total**	10,760 (100%)	4,973 (46.2%)	3,694 (34.3%)	2,093 (19.5%)	
**Sex**					<0.001
Male	5,872 (54.6%)	2,246 (45.2%)	2,156 (58.4%)	1,470 (70.2%)	
Female	4,888 (45.4%)	2,727 (54.8%)	1,538 (41.6%)	623 (29.8%)	
**Age**					<0.001
20–29	1,447 (13.4%)	676 (13.6%)	556 (15.1%)	215 (10.3%)	
30–39	2,807 (26.1%)	1,228 (24.7%)	1,061 (28.7%)	518 (24.7%)	
40–49	3,004 (27.9%)	1,435 (28.9%)	1,027 (27.8%)	542 (25.9%)	
50–59	2,243 (20.8%)	1,048 (21.1%)	723 (19.6%)	472 (22.6%)	
60 and older	1,259 (11.7%)	586 (11.8%)	327 (8.9%)	346 (16.5%)	
**Household income**					<0.001
Low	7,891 (73.3%)	3,718 (74.8%)	2,785 (75.4%)	1,388 (66.3%)	
High	2,869 (26.7%)	1,255 (25.2%)	909 (24.6%)	705 (33.7%)	
**Education**					<0.001
Under highschool	4,977 (46.3%)	2,114 (42.5%)	1,613 (43.7%)	1,250 (59.7%)	
University	5,783 (53.7%)	2,859 (57.5%)	2,081 (56.3%)	843 (40.3%)	
**Body mass index**					<0.001
Normal	7,131 (66.3%)	3,422 (68.8%)	2,430 (65.8%)	1,279 (61.1%)	
Overweight or obese	3,629 (33.7%)	1,551 (31.2%)	1,264 (34.2%)	814 (38.9%)	
**Drinking**					<0.001
Non-drinker	8,790 (81.7%)	4,206 (84.6%)	2,990 (80.9%)	1,594 (76.2%)	
Drinker	1,970 (18.3%)	767 (15.4%)	704 (19.1%)	499 (23.8%)	
**Smoking**					<0.001
Non smoker	5,706 (53.0%)	2,996 (60.2%)	1,889 (51.1%)	821 (39.2%)	
Past smoker	2,504 (23.3%)	1,076 (21.6%)	878 (23.8%)	550 (26.3%)	
Current smoker	2,550 (23.7%)	901 (18.1%)	927 (25.1%)	722 (34.5%)	
**Occupation group**					<0.001
Manual worker	3,472 (32.3%)	1,316 (26.5%)	1,182 (32.0%)	974 (46.5%)	
Non-manual worker	7,288 (67.7%)	3,657 (73.5%)	2,512 (68.0%)	1,119 (53.5%)	
**Employment status**					<0.001
Full time job	9,083 (84.4%)	4,231 (85.1%)	3,179 (86.1%)	1,673 (79.9%)	
Temporary job	1,245 (11.6%)	553 (11.1%)	398 (10.8%)	294 (14.0%)	
Day labor	432 (4.0%)	189 (3.8%)	117 (3.2%)	126 (6.0%)	
**Shiftwork**					<0.001
Daywork	9,312 (86.5%)	4,449 (89.5%)	3,221 (87.2%)	1642 (78.5%)	
Shiftwork	1,448 (13.5%)	524 (10.5%)	473 (12.8%)	451 (21.5%)	
**Dining out**					<0.001
Less than once a week	1,169 (10.9%)	532 (10.7%)	314 (8.5%)	323 (15.5%)	
1–6 times a week	4,951 (46.1%)	2,508 (50.5%)	1,667 (45.2%)	776 (37.3%)	
More than once a day	4,613 (43.0%)	1,922 (38.7%)	1,707 (46.3%)	984 (47.2%)	

The mean daily fiber intake of the total population was 25.1 ± 13.4 grams. Mean fiber per 1,000 kcal (daily energy intake) was 12.2 ± 5.4 grams. The majority (70.1%) of the total population had an insufficient intake of dietary fibers. When working hour groups were compared, the total amount of dietary fiber was consumed the most by the group working for more than 52 h per week. However, the amount of fiber per 1,000 kcal was the lowest in the group working for more than 52 h per week. As a result, the prevalence of fiber insufficiency was the highest in the group working for more than 52 h per week (Table 2).

**Table 2:** Difference of dietary fiber intake according to working hours per week.

	**Total population**	**Working hours per week**			
		**30–40 h**	**41–52 h**	**>52 h**	***P*** **-value**
	***N*** **(%)**	***N*** **(%)**	***N*** **(%)**	***N*** **(%)**	
**Fiber (g)**	25.1 ± 13.4	24.9 ± 13.4	25.1 ± 13.2	25.2 ± 13.4	0.591
**Energy (kcal)**	2168.5 ± 962.1	2080.1 ± 916.2	2227.6 ± 980.3	2274.1 ± 1016.5	<0.001
**Fiber per energy (g/kcal)**	12.2 ± 5.4	12.6 ± 5.6	11.8 ± 5.2	11.7 ± 5.3	<0.001
**Fiber insufficiency**					<0.001
Sufficient (≥14 g/kcal)	3217 (29.9%)	1649 (33.2%)	1008 (27.3%)	560 (26.8%)	
Insufficient (<14 g/kcal)	7543 (70.1%)	3324 (66.8%)	2686 (72.7%)	1533 (73.2%)	

To identify covariates showing statistically significant associations with fiber insufficiency, simple logistic regression analysis was performed. In all age groups, males (OR: 1.61, 95% CI: 1.46–1.77), drinkers (OR: 1.98, 95% CI: 1.73–2.28), past smokers (OR: 1.19, 95% CI: 1.06–1.34), current smokers (OR: 2.27, 95% CI: 1.99–2.58), university graduates (OR: 1.3, 95% CI: 1.19- 1.44), working FOR 41–52 h per week (OR; 1.32, 95% CI: 1.18–1.47), and working for more than 52 h per week (OR: 1.42, 95% CI: 1.25–1.62) were significantly associated with insufficient fiber intake (Table 3). The risk of dietary fiber deprivation varied greatly among age groups. In particular, the risk of dietary fiber shortage in the age group of 20s (OR: 6.77, 95% CI: 5.46–8.41) was six times higher than that for those in their 60s as a reference group. Because of such discrepancy by age groups, we stratified subjects by age groups. In the group of 20s, only current smokers (OR: 2.55, 95% CI: 1.54–4.23) were associated with fiber insufficiency. Working for more than 52 h per week was also associated with fiber insufficiency (Table 3).

**Table 3:** Logistic regression models for dietary fiber insufficiency (<14 g/kcal per day).

	**Proportion of fiber insufficiency (<14 g/kcal)**	**Crude model**		Adjusted model^*^	
		**Odds ratio**	**95% confidence interval**	**Odds ratio**	**95% confidence interval**
**Sex**					
Male	74.8%	(Ref)		(Ref)	
Female	64.4%	1.61	1.46–1.77	1.29	1.12–1.48
**Age**					
20–29	87.5%	6.77	5.46–8.41	8.55	6.70–10.92
30–39	81.4%	4.44	3.76–5.24	5.04	4.17–6.11
40–49	70.8%	2.58	2.21–3.02	2.90	2.44–3.44
50–59	54.7%	1.23	1.05–1.44	1.31	1.11–1.55
60 and older	50.6%	(Ref)		(Ref)	
**Household income**					
Low	69.5%	(Ref)		(Ref)	
High	70.3%	1.07	0.96–1.19	1.12	0.99–1.26
**Education**					
Under high school	66.2%	(Ref)		(Ref)	
University	73.4%	1.3	1.19–1.44	0.93	0.82–1.06
**BMI**					
Normal	69.7%	(Ref)		(Ref)	
Overweight or obese	70.9%	1.01	0.92–1.12	0.93	0.83–1.04
**Drinking**					
Non-drinker	67.5%	(Ref)		(Ref)	
Drinker	81.7%	1.98	1.73–2.28	1.63	1.4–1.9
**Smoking**					
Nonsmoker	65.0%	(Ref)		(Ref)	
Past smoker	70.2%	1.19	1.06–1.34	1.16	1.00–1.36
Current smoker	81.4%	2.27	1.99–2.58	1.63	1.38–1.92
**Occupation group**					
Manual worker	67.6%	(Ref)		(Ref)	
Non-manual worker	71.3%	1.09	0.98–1.20	0.88	0.77–1.00
**Employment status**					
Full time job	70.3%	(Ref)		(Ref)	
Temporary job	69,8%	1.04	0.89–1.21	0.93	0.79–1.11
Daily labor	67.6%	1.08	0.85–1.37	1.35	1.04–1.76
**Shiftwork**					
Day work	70.3%	(Ref)		(Ref)	
Shiftwork	68.7%	0.99	0.86–1.14	0.9	0.77–1.05
**Weekly working hours**					
30h−40h	66.8%	(Ref)		(Ref)	
41h−52h	72.7%	1.32	1.18–1.47	1.14	1.02–1.28
52h<	73.2%	1.42	1.25–1.62	1.27	1.10–1.47

* *Adjusted for sex, body mass index, occupation, drinking, smoking, household income, education, and shiftwork*.

Multiple logistic regression model was established after adjusting for gender, age groups, job groups, drinking habits, smoking habits, and education that showed significant associations with fiber insufficiency in simple logistic regression analysis. After adjustment, compared to the group working for <40 h per week, the group working for 41–52 h per week (OR: 1.13, 95% CI: 1.01–1.27) and the group working for more than 52 h per week (OR: 1.26, 95% CI: 1.09–1.45) had higher risk of fiber insufficiency. After stratifying subjects into different age groups, the same trend was observed for all age groups except for the age group of 20s or 30s (Table 4).

**Table 4:** Logistic regression models for dietary fiber insufficiency (<14 g/kcal per day).

	**Weekly working hours**	**Proportion of fiber insufficiency (<14 g/kcal)**	Odds ratio^*^	**95% confidence interval**	***P*** **for trend**
**All age**					
	30–40 h	66.8%	(Ref)		0.001
	41–52 h	72.7%	1.14	1.02–1.28	
	>52 h	73.2%	1.27	1.10–1.47	
**Age 20s**					
	30–40 h	86.1%	(Ref)		0.414
	41–52 h	88.5%	1.32	0.89–1.93	
	>52 h	89.3%	1.09	0.63–1.91	
**Age 30s**					
	30–40 h	80.0%	(Ref)		0.379
	41–52 h	81.2%	0.94	0.74–1.2	
	>52 h	84.9%	1.22	0.88–1.69	
**Age 40s**					
	30–40 h	66.8%	(Ref)		0.185
	41–52 h	74.1%	1.25	1.01–1.54	
	>52 h	75.3%	1.12	0.86–1.46	
**Age 50s**					
	30–40 h	50.8%	(Ref)		0.033
	41–52 h	55.9%	1.09	0.87–1.36	
	>52 h	61.7%	1.34	1.03–1.74	
**Age 60s or older**					
	30–40 h	45.7%	(Ref)		0.007
	41–52 h	51.1%	1.19	0.87–1.64	
	>52 h	58.4%	1.55	1.13–2.12	

* *All stratified models were adjusted for sex, body mass index, occupation, drinking, smoking, household income, education, and shiftwork*.

## Discussion

In this study, we found an association between long working hours and daily dietary fiber intake. This association was especially significant in older age groups, while relatively younger age groups (20s, 30s, and 40s) did not show such association between long working hours and dietary fiber intake.

Increasing working hours inevitably reduce leisure time, which tends to reduce unpaid work to exploit this limited leisure time (24). According to an OECD report in 2014, Korean people spent 1.4 h per day for routine household works, including cooking, cleaning, and home maintenance (25). Lack of time spent for preparing and cooking might be related to an unhealthy diet pattern (26). In our study, 47.2% of people who work for more than 52 h per week eat out daily.

If people don't have much time to rest, they are tended to eat out rather than cook at home. Eating out behavior tend to intake higher energy, fat, and sodium, but lower fruits and vegetables, which contain rich of fiber. In the previous study, there was even association between frequency of eating away and risk of all causes of mortality rate (27). For the lack of time, working people tend to eat ready-made meals or convenience food. According to the previous study, Korean people intake more calories from these foods than 20 years ago (28). These foods also tend to have less fiber and higher fat, sodium, and energy (28, 29).

In early researches, dietary fiber was known to be associated with numerous diseases and abnormal conditions such as simple constipation, diverticulitis, and even possible colon carcinoma (30). Moreover, because dietary fiber can act as a vehicle for fecal water by increasing fecal volume, (2) it is important for preventing colorectal disorders, especially adenomas and carcinomas. However, systematic reviews did not draw conclusive evidence that dietary fiber could prevent them (31, 32). A few studies have suggested an association between working time and colorectal cancer. In a case-control study conducted in Spain, workers who had ever performed a rotating shift work showed an increased risk of colorectal cancer compared to daytime workers (33). Also, a multicenter cohort has reported a hazard ratio of 1.41 of working for 55 h per week or more compared to workers who work for 35–40 h per week, although the result is not statistically significant (34). Suggested mechanisms involved in the association of long working time with colorectal cancer include hormonal effects mainly. However, results of the current study suggest another mechanism via distortion of dietary habits by working hours.

Besides health conditions regarding colon, hormonal effects of dietary fiber have been emphasized. Glycemic index is a major index for glucose metabolism. Generally, foods that contain high dietary fiber have small glycemic indices. A low glycemic index meal rich in dietary fiber can improve glucose tolerance (35). Therefore, consistent dietary fiber intake is associated with low risk of metabolic syndrome, although evidences from cohort data are insufficient (36, 37). Long working hours are well-known as a risk factor for cardiovascular diseases. Large cohort studies and systematic reviews have consistently reported the relationship of cardiovascular diseases with long working hours (4, 6, 16). It is mainly explained by the fact that working for a long time affects sympathetic nervous activity or accelerates thrombotic reaction (38, 39). Some studies with long working hours have shown the elevation of high-sensitivity C-reactive protein, which is a risk factor of cardiovascular disease via systematic inflammation (40, 41). A recent study has shown that dietary fiber intake could lower the risk of other disorders such as non-alcoholic fatty liver disease and hyperuricemia (42, 43).

This study emphasized the association between working hours and dietary fiber intake, showing significant differences by age groups. We could only observe the association of long working hours with dietary fiber intake in relatively old age groups in stratified analyses because effects from age were much greater than those from working hours. We assume that there is not only an age effect, but also a cohort effect, meaning that the difference might be caused by participants' birth year (thus the generation). For example, dietary habits of people who were born in the 1960s and those of people who were born in the 1990s might be totally different because of the generational difference in dietary habits. Furthermore, because Korea achieved economic growth rapidly, the association could be more exaggerated in Korea than in other countries. However, few studies have discussed this topic. Therefore, more detailed analyses about the age-cohort effect on dietary fiber intake are needed in the future.

Because this study used nation-wide representative data, we could expect robustness of statistical analyses. Qualified dietary food fiber calculated by results from face-to-face interviews by trained surveyors in a large sample was a major strength of this study. However, because of its cross-sectional design, results of this study could not guarantee a causal relationship that working for long hours caused dietary fiber insufficiency. However, it is difficult to present a possibility that dietary fiber insufficiency could cause working for long hours. Because of this limitation, we could not firmly state that there is a causal relationship between long working hours and a high fiber insufficiency rate. In further research, a panel study with focus group interviews could be helpful. Confounding factors such as economic status, educational level, and other health behaviors might affect dietary fiber intake. However, our multiple regression analysis model was robust after adjusting for potential confounders.

This is the first study suggesting that workers with long working hours could have problems of health behaviors due to dietary fiber insufficiency. Thus, professionals in occupational health should focus on dietary habits of workers with long working hours. Moreover, more detailed studies dealing with causal factors for dietary fiber insufficiency in workers with long working hours are needed.

## Data Availability Statement

The original contributions presented in the study are included in the article/supplementary materials, further inquiries can be directed to the corresponding author.

## Ethics Statement

The studies involving human participants were reviewed and approved by Institutional Review Board of St. Mary's Hospital, Catholic University of Korea. The patients/participants provided their written informed consent to participate in this study.

## Author Contributions

JM and JL conceptualized, established the design of the study, and wrote the manuscript. D-WL and M-YK analyzed and interpreted data. J-PM and H-RK interpreted data and revised the manuscript critically. All authors contributed to the article and approved the submitted version.

## Conflict of Interest

The authors declare that the research was conducted in the absence of any commercial or financial relationships that could be construed as a potential conflict of interest.

## Publisher's Note

All claims expressed in this article are solely those of the authors and do not necessarily represent those of their affiliated organizations, or those of the publisher, the editors and the reviewers. Any product that may be evaluated in this article, or claim that may be made by its manufacturer, is not guaranteed or endorsed by the publisher.
